# Feasibility and acceptability of electronic symptom surveillance with clinician feedback using the Patient-Reported Outcomes version of Common Terminology Criteria for Adverse Events (PRO-CTCAE) in Danish prostate cancer patients

**DOI:** 10.1186/s41687-017-0005-6

**Published:** 2017-09-12

**Authors:** Christina Baeksted, Helle Pappot, Aase Nissen, Niels Henrik Hjollund, Sandra A. Mitchell, Ethan Basch, Pernille Envold Bidstrup, Susanne Oksbjerg Dalton, Christoffer Johansen

**Affiliations:** 1 Documentation & Quality, Danish Cancer Society, Strandboulevarden 49, DK-2100 Copenhagen, Denmark; 2 Department of Oncology, The Finsen Centre, Rigshospitalet, Blegdamsvej 9, DK-2100 Copenhagen, Denmark; 3 Department of Clinical Epidemiology, Aarhus University Hospital, Olof Palmes Allé 43-45, DK-8200 Aarhus, Denmark; 4 WestChronic, Department of Occupational Medicine, University Clinic, Health, Aarhus University, Gl. Landevej 61, DK-7400 Herning, Denmark; 5 Outcomes Research Branch, Division of Cancer Control and Population Sciences, National Cancer Institute, 9609 Medical Center Drive, 3 East Suite 448, Rockville, MD 20850 USA; 6 Cancer Outcomes Research Program, UNC Lineberger Comprehensive Cancer Center, 101 Manning Dr, Chapel Hill, NC 27514 USA; 7 Unit of Survivorship, Danish Cancer Society Research Center, Strandboulevarden 49, DK-2100 Copenhagen, Denmark

**Keywords:** PRO-CTCAE, Feasibility, Prostate cancer, Symptom surveillance, Electronic reporting

## Abstract

**Background:**

The aim was to examine the feasibility, acceptability and clinical utility of electronic symptom surveillance with clinician feedback using a subset of items drawn from the Patient-Reported Outcomes version of Common Terminology Criteria for Adverse Events (PRO-CTCAE) in a cancer treatment setting.

**Methods:**

Danish-speaking men with castration-resistant metastatic prostate cancer receiving treatment at the Department of Oncology, Rigshospitalet, Copenhagen between March 9, 2015 and June 8, 2015 were invited to participate (*n* = 63 eligible). Participants completed the PRO-CTCAE questionnaire on tablet computers using AmbuFlex software at each treatment visit in the outpatient clinic. In total, 22 symptomatic toxicities (41 PRO-CTCAE items), corresponding to the symptomatic adverse-events profile associated with the regimens commonly used for prostate cancer treatment (Docetaxel, Cabazitaxel, Abiraterone, Alpharadin), were selected. Participants’ PRO-CTCAE responses were presented graphically to their treating oncologists via an AmbuFlex dashboard, for real-time use to enhance the patient-clinician dialogue that occurs during the consultation prior to each treatment cycle. Technical and clinical barriers and acceptability were evaluated through semi-structured interviews with both patients and oncologists. Patients receiving active treatment at the end of the study period completed an evaluation questionnaire.

**Results:**

Fifty-four out of sixty-three (86%) eligible patients were enrolled. The PRO-CTCAE questionnaire was completed a total of 168 times by 54 participants (median number per patient was 3, range 1–5). Eight surveys were missed, resulting in a compliance rate of 97%. At the end of the study period, 35 patients (65%) were still receiving active treatment and completed the evaluation questionnaire. Patients reported that their PRO-CTCAE responses served as a communication tool. Oncologists stated that the availability of the PRO-CTCAE self-reports during the consultation improved patient-clinician communication about side effects.

**Conclusion:**

Electronic capture of symptomatic toxicities using PRO-CTCAE and the submission of self-reports to clinicians prior to consultation were feasible among metastatic prostate cancer patients receiving chemotherapy in an outpatient setting, and this procedure was acceptable to both patients and clinicians. Continued research, including a cluster-randomized trial, will evaluate the effects of submitting patients’ PRO-CTCAE results to clinicians prior to consultation on the quality of side-effects management and resultant clinical outcomes.

## Background

Serial evaluations of treatment tolerability are an important component of clinical monitoring of cancer therapy [1]. Tolerability determinations are based on physical examination, laboratory testing and patient self-reporting [1]. In cancer treatment, patients are unsystematically asked about toxicity symptoms, and the severity and presence of these symptoms are interpreted and registered by clinicians. Further, observational studies comparing the reporting of symptoms via clinicians versus direct patient self-reporting have found that clinicians’ ratings of symptom severity are lower than those gathered using patient-reported outcome measures [2–5]. Thus, capturing symptomatic toxicities directly from patients using patient-reported outcome measures may improve the accuracy of identifying and characterizing the symptomatic adverse effects of treatment [6, 7].

Now, version 4 of the National Cancer Institute’s Common Terminology Criteria for Adverse Events (CTCAE) is the standard for reporting adverse events in cancer clinical trials and is widely used in cancer treatment and research settings [7]. As a companion to the CTCAE, NCI has developed a Patient-Reported Outcome version of the Common Terminology Criteria for Adverse Events (PRO-CTCAE) [8].

Studies examining the feasibility of electronic patient-reporting of symptomatic side effects of cancer treatment have shown high acceptability of self-reporting presented as high satisfaction with using the system [9–12], and the patient’s experience of aided recall of symptoms helped them feel more in control of their care [12]. Prior studies have used hardcopy feedback reports to clinicians or a limited number of symptom questions, as well as a variety of software systems for patient reporting [9–14]. In this study, we aimed to use a questionnaire covering all the relevant symptomatic toxicities for a specific cancer treatment, which is important in order to use the patient’s reporting in the discussion of treatment tolerance and supportive care with the oncologist. Additionally, patients’ electronic reporting of side effects prior to chemotherapy could be displayed in real-time to both clinicians and patients using an established patient-reported outcome (PRO) dashboard [15]. This makes it possible to incorporate patients’ experiences into the treatment-related communication and decision-making, including side-effects management and supportive care. We have recently translated and linguistically validated a Danish language version of the full PRO-CTCAE library [16]. In this, we report the results of a pilot study examining the feasibility and acceptability to patients and clinicians of collecting these data in routine clinical practice with prostate cancer patients using PRO-CTCAE-Danish.

The purpose of this study was to examine the feasibility, acceptability and clinical utility among patients with prostate cancer and their oncologists of systematic electronic Patient-Reported Outcome (PRO) data collection using a subset of PRO-CTCAE items, with feedback to clinicians in real-time during active treatment. Patients included were castration-resistant metastatic prostate cancer patients receiving medical oncology treatments in an oncology clinic during a three-month study period. This is the first study in a Danish cancer treatment setting to investigate the feasibility of using a treatment-specific subset of PRO-CTCAE items in a specific patient group during active cancer treatment.

## Methods

### Study population

Male patients being treated for castration-resistant metastatic prostate cancer, who were able to read, write, and speak Danish, and who were receiving chemotherapy at Department of Oncology, Rigshospitalet, Copenhagen University Hospital from March 9, 2015 to June 8, 2015 were invited to participate in this study. The participants were either initiating treatment, receiving ongoing treatment or transitioning to a new treatment regimen. All participants received oral and written information about the study, and they gave their informed consent for participation.

### PRO-CTCAE

Items from PRO-CTCAE corresponding to the symptomatic adverse-events profile associated with systemic treatment of prostate cancer using Docetaxel, Cabazitaxel, Abiraterone or Alpharadin in phase III clinical, randomized trials were used in this study [17–20]. This resulted in 41 PRO-CTCAE items (presence, amount, frequency, severity and/or interference with daily activities) reflecting 22 symptomatic toxicities that were assessed at each clinic visit. The recall period for PRO-CTCAE is the past seven days [8]. Conditional branching was not employed, which means that patients were presented with all items regardless of their answers. If the patients experienced symptoms not covered by the 41 PRO-CTCAE items, additional symptoms were solicited using the free-text, write-in feature, as shown in Table 1.

**Table 1:** Twenty-two PRO-CTCAE symptom terms (41 items) and a write-in text box

PRO-CTCAE symptom term	Presence/absence	Amount	Frequency	Severity	Interference with daily activities
Watery eyes				x	x
Abdominal pain			x	x	x
Constipation				x	
Diarrhea			x		
Difficulty swallowing				x	
Nausea			x	x	
Vomiting			x	x	
Fatigue				x	x
General pain			x	x	x
Decreased appetite				x	x
Joint pain			x	x	x
Muscle pain			x	x	x
Taste changes				x	
Numbness & tingling				x	x
Cough				x	x
Shortness of breath				x	x
Nosebleed			x	x	
Hair loss		x			
Nail discoloration	x				
Nail ridging	x				
Rash	x				
Swelling			x	x	x
	Free-text, write-in feature				
Other symptoms					

### AmbuFlex

**Fig. 1:**
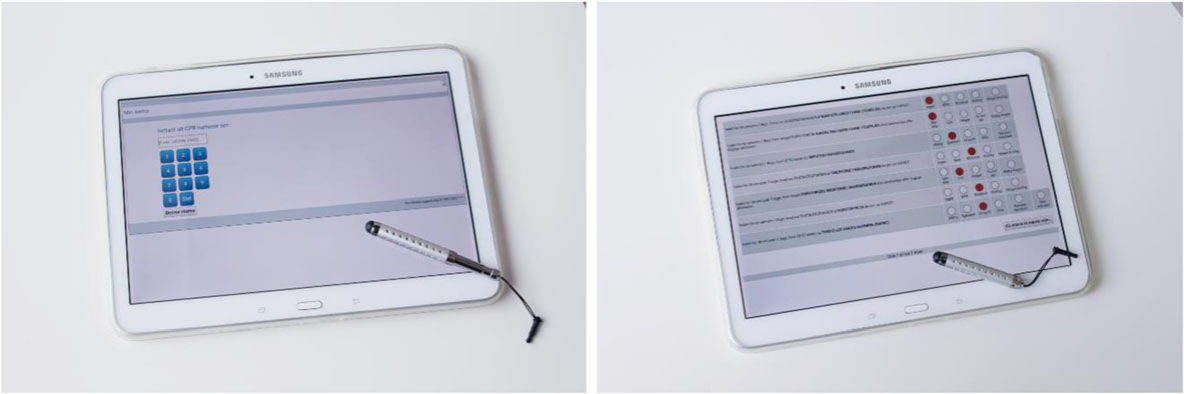
Patients log on to the tablet computer using the unique civil registration number and complete the PRO-CTCAE questionnaire in the waiting room

**Fig. 2:**
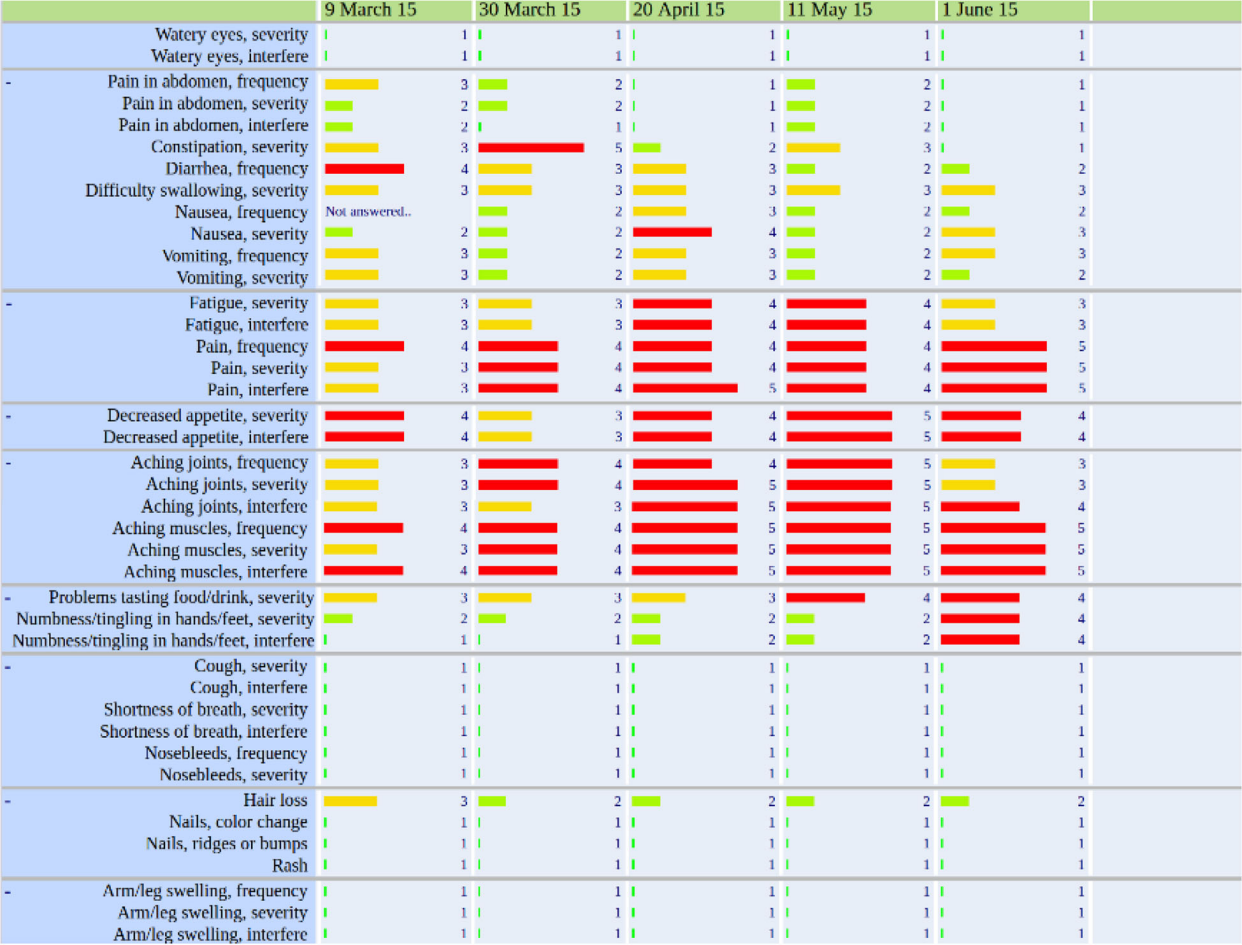
Overview of patients’ reporting available at the oncologists’ computers in real-time. Items are presented as bars with different colors (for example, red = very severe, orange = severe, yellow = moderate, light green = mild, dark green = none), lengths and numbers (1–5) for each date of treatment visit. Note: In our study, the symptomatic toxicities were in Danish language, but are here presented in English

A total of 41 PRO-CTCAE items selected for this study were encoded in the AmbuFlex software and presented to patients on tablet computers provided at the clinic (Fig. 1). AmbuFlex software has been developed and used in Denmark to collect patient-reported outcomes in a variety of chronic diseases [15, 21]. In the AmbuFlex software, the patients’ response was available as a graphic real-time presentation on the oncologists’ computer screen. Scores for each item were reported as bars with assorted colors, lengths and numbers (Fig. 2). Presentation of graphics from previous visits alongside the most recent response made it possible to identify change over time. At patient visits, output was available on the oncologist’s computer screen, which made it possible to discuss the experienced symptoms with the patient. The AmbuFlex software was integrated into the software system clinicians use to order systemic anticancer treatments, including chemotherapy.

### Procedures for patient reporting and feedback to the oncologist

1. Before the first treatment with chemotherapy, patients were given a 5–10-min introduction to the tablet computer, its functionality and how to complete the questionnaire. The patients completed the PRO-CTCAE questionnaire on the tablet computer in the waiting room, using their unique personal identification number, which all Danish citizens are assigned at birth by the Danish Civil Registration System (CRS) [22], as their logon to the tablet computer (Fig. 1). Patients were asked to complete the PRO-CTCAE questionnaire at each treatment visit every third week in the clinic prior to consultation with the oncologist. Throughout the entire study period, support was provided on site by the researcher.
2. Each oncologist received a brief (5–10 min) individual training session from the researcher focused on how to navigate the AmbuFlex software system, including how to locate and interpret the graphical depictions of PRO-CTCAE scores provided by the AmbuFlex web system.
3. The oncologists were encouraged to review the PRO-CTCAE responses before meeting the patient or while talking with the patient, but were not obliged to do so as the electronic patient reporting was not incorporated into local guidelines. At weekly clinic meetings, the oncologists were informed about the progress of the study and any logistical issues (information about the tablet computers, possible technical issues, technical support, etc.) arising during daily practice.

### Semi-structured interviews and questionnaire data collection

Technical feasibility, acceptability and clinical utility were evaluated by combining individual semi-structured interviews with patients and oncologists, and by means of an evaluation questionnaire (developed by the researchers) for patients. The three themes—technical feasibility, acceptability and clinical utility—were selected based on lessons learned from other studies involving electronic patient reporting of symptoms [11, 12]. The researcher registered information on participation and technical problems on a daily basis. Additionally, a log was recorded from AmbuFlex showing the number of times that patients’ completions of PRO-CTCAE questionnaire were reviewed in AmbuFlex by an oncologist.

- Eight weeks into the study period, four patients were interviewed individually by one researcher about their experiences of completing the PRO-CTCAE questionnaire on the tablets and their experience of the use of their responses by the oncologists. These semi-structured interviews were used to identify relevant questions within the three themes of importance to the patients. Based on these interviews, an evaluation questionnaire was developed. The interviews aimed to develop the evaluation questionnaire and to present detailed information.
- All participants receiving treatment in the clinic during the last three weeks of the study period were asked to complete the evaluation questionnaire (*n* = 35). The evaluation questionnaire comprised 13 items regarding the patients’ experience of completing the PRO-CTCAE questionnaire on tablets in the clinic (information and help with technical issues, waiting area, oncologists’ and nurses’ use of patients’ reporting). Additionally, there were two items on the patients’ experience of using tablets and computer/internet in their daily life, four items on age, occupation, highest attained education and marital status, and a blank write-in field for other comments. The results were summarized using descriptive statistics.
- At the end of the study period, one researcher conducted individual semi-structured interviews with the five oncologists treating prostate cancer patients in the clinic about their opinions and experiences of using the patients’ self-reporting of symptoms during the clinical encounter.

The researcher conducted the semi-structured interviews in accordance with interview guides (five questions in the interview guide for patients and seven questions in the interview guide for oncologists), focusing on the themes technical feasibility, acceptability and clinical utility. All interview data was analyzed for content and summarized according to the above-mentioned themes. The interview data is presented with quotations from patients and oncologists. Quotations were selected to cover both facilitators and barriers.

## Results

### Participation

Between March 9, 2015 and June 8, 2015, 60 out of 63 eligible patients were approached to participate in the study and 54 enrolled, resulting in a participation rate of 90% (54/60). The median age of the participants was 69 years (range 51–88 years). The reasons for declining to participate included fatigue (*n* = 3) and a lack of interest in the study (*n* = 3), while three patients were not invited due to logistical problems (for example, the researcher was not in the clinic when the patient met for treatment).

### Feasibility of in-clinic PRO-CTCAE patient reporting with feedback to treating oncologist

The PRO-CTCAE questionnaire was completed a total of 168 times by 54 participants (Table 2). Eight patients received help using the screen on the tablet computer from their spouse, other relative or the researcher. The problem was for all patients related to sensitivity of the touch screen. A log from AmbuFlex showed that 45 of the 54 patients (83%) had at least one PRO-CTCAE questionnaire reviewed by the oncologist during the study period. Of the 168 completions of questionnaire, 87 completions (52%) were reviewed by an oncologist.

**Table 2:** Number of patients completing PRO-CTCAE

	First PRO-CTCAE completion	Second PRO-CTCAE completion	Third PRO-CTCAE completion	Fourth PRO-CTCAE completion	Fifth PRO-CTCAE completion	Total number of completions and missed completions
Number of patients completing PRO-CTCAE	52	50	34	24	8	168
Number of patients with missed completion	2	3	0	1	0	6

Six patients missed one completion of the scheduled 174 responses of the PRO-CTCAE questionnaire, giving a compliance rate of 97% (Table 2). The reasons for missing data included late arrival to the clinic (*n* = 4), difficulty logging onto the system (*n* = 1), and being too ill to report (*n* = 1). Furthermore, during the study period, three patients once completed the PRO-CTCAE questionnaire after their consultation with the oncologist.

### Patient interviews and evaluation questionnaire

Four patients were individually interviewed, and the themes important to patients and the patients’ contribution were incorporated into the evaluation questionnaire. After interviewing four patients, a point of information saturation was reached. Since the evaluation questionnaire was based on information from some of the first patients included in the study, the questionnaire was only available for patients in active treatment in the last three weeks of the study period. Thirty-five patients (100% of the patients invited to complete the evaluation questionnaire, 65% of the study population) responded to the evaluation questionnaire. Their median age was 69 years, and 37% had basic/high school or vocational education as their highest attained education (Table 3).

**Table 3:** Characteristics of patients who completed the structured evaluation questionnaire (*n* = 35)

Median age (range) in years	69 (56–79)
Highest attained education	*n* (%)
Basic or high school	4 (11)
Vocational education	9 (26)
Higher education, 2–4 years	9 (26)
Higher education, ≥ 5 years	6 (17)
Unknown	7 (20)
Employment status	
Working full time	5 (14)
Working part time	1 (3)
Retired	22 (63)
Unknown	7 (20)
Marital status	
Single	3 (9)
Married or cohabiting	24 (69)
Divorced or separated	5 (14)
Unknown	3 (9)
Frequency of Use of Internet/Computer	
Never	1 (3)
Sometimes	6 (17)
Often	23 (66)
Missing	5 (14)
Tablet computer or smartphone at home	
No	8 (23)
Yes	23 (66)
Missing	4 (11)

#### Technical feasibility

Patients found the tablet computers easy to use. However, 23% of the patients needed help at least for the first time they used it (Table 4). Data from AmbuFlex software showed that the mean time for completing the PRO-CTCAE questionnaire was 6 min and 48 s (ranging from 3 min and 3 s to 46 min and 24 s). Six patients reported technical problems (Table 4), such as the tablet computer shutting down and having to start over or a slow responding tablet computer and having to touch each key more than once. Three of the six patients reporting technical problems are part of the 23% who needed help using the tablet computer.

**Table 4:** Results from the patient evaluation questionnaire (*n* = 35)

What was your experience of using the tablet computer	*n* (%)
Very easy	24 (69)
Easy	10 (29)
Difficult/Very Difficult	1 (3)
Did you need any help to use the tablet computer	
No	26 (74)
Only the first time	7 (20)
On several occasions	1 (3)
Missing	1 (3)
Did you get the help you needed (*n* = 8)	
Yes, from relatives	5 (63)
Yes, from the researcher	7 (88)
No, I did not get the help I needed	0 (0)
Did you experience any technical problems	
No	28 (80)
Yes	6 (17)
Missing	1 (3)
How did you experience your symptom reporting was used in the clinic^a^	
The oncologist had reviewed my symptom reporting before the consultation	16 (46)
The oncologist talked with me about my symptom reporting	12 (34)
The nurse had reviewed my symptom reporting	4 (11)
I don’t think that neither the oncologist nor the nurse had reviewed my symptom reporting	4 (11)
Do not know	9 (26)
The electronic PRO-CTCAE items provided a complete picture of my symptomatic side effects	
Totally agree	5 (14)
Agree	22 (63)
Neither agree nor disagree	2 (6)
Disagree/totally disagree	0 (0)
Do not know	1 (3)
Missing	5 (14)
The electronic symptom reporting is a good tool when talking with the oncologist	
Totally agree	5 (14)
Agree	22 (63)
Neither agree nor disagree	1 (3)
Disagree/totally disagree	(0)
Do not know	3 (9)
Missing	4 (11)
The electronic symptom reporting results in more focus on side effects in the consultation	
Totally agree	3 (9)
Agree	11 (31)
Neither agree nor disagree	9 (26)
Disagree/totally disagree	1 (3)
Do not know	6 (17)
Missing	5 (14)

Some questionnaires were returned without full completions

^a^More than one statement could be ticked

#### Clinical utility

A total of 46% of patients perceived that the oncologist had reviewed their self-reported data in AmbuFlex prior to the clinical encounter, and 34% of patients reported that their oncologist talked with them about their self-reported symptoms during the clinic visit (Table 4). One patient explained: *“The dialogue with the oncologist is more efficient. The questions are about relevant problems. You can see where symptoms differ from the last treatment, such as new side effects”* and another patient explained: *“First the doctor asked about my answers: “You have stated that …”.*

#### Acceptability

A total of 40% of patients reported more focus on side effects compared to their prior treatments in the consultation with the oncologist after answering the PRO-CTCAE questions and 77% thought it was a useful tool when talking to the oncologist (Table 4). Most patients (77%) stated that the PRO-CTCAE questionnaire enabled them to provide a complete picture of their side effects (Table 4). One patient explained: *“You are the one who knows best and feels the side effects. You can give a more complete picture.”* Another stated: *“You remember things to talk about with the doctor.”* The system also helped patients be more aware of their symptoms, as explained by a patient: *“You become more aware of what happens to you. You get an opportunity to follow the changes in the symptoms.”*

### Oncologist interviews

#### Technical feasibility

The five oncologists found the AmbuFlex software easy to use and did not spend more time than usual on the consultation when using the software. However, one oncologist mentioned some concern about this new task: *“It is a barrier that you have to log in to another system [AmbuFlex] if it does not substitute other tasks.”* Four out of five oncologists described the graphic presentation as clear and providing a good overview, while one oncologist suggested adding more pictures and/or graphs of the patients’ symptoms. As there are continuously new oncologists in training in the clinic, one of the oncologists was given the introduction to the system after he had started using it: *“I came to the department after the project had started. I had some questions about how to use the system.”*

#### Clinical utility

The oncologists expressed different views on the clinical use of the patients’ reporting of PRO-CTCAE symptoms. Two oncologists expressed concern regarding the patients’ rating of symptoms. One oncologist explained: “*The patient’s self-reporting gives a picture of the patient’s overall condition, but the patient’s answers were not necessarily related to the chemotherapy.*” Another expressed that “*Patients rate their symptoms differently than we [the oncologists – ed.] would do, and patients often underestimate or overestimate the severity of their symptoms.*” In addition, positive comments were reported as expressed by one oncologist: *“You get a better impression of the patient’s overall situation—symptoms and side effects.”* Patient reporting was generally seen by oncologists as a supplement to the clinical dialogue with the patient in the consultation. For example, one oncologist said*: “It is positive that the patients rate themselves—sometimes we underestimate. But the dialogue is important for specifying the [symptom and severity].”*

#### Acceptability

The oncologists’ general experience was expressed by one who said: *“The patients are better prepared and have thought about the symptoms they have experienced since the last visit,”* when the patients have answered the PRO-CTCAE questionnaire on the tablet computer before the consultation. However, one oncologist stated that patients could find it difficult to separate the gradings ‘severity’ and ‘influence on daily activity’: *“Too many similar questions. But the right thing to do—for both patients and doctors.”*

**Fig. 3:**
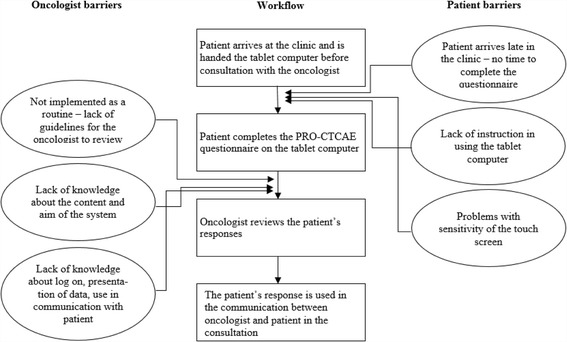
Workflow and barriers for oncologists and patients in using electronic patient reporting of symptomatic toxicities

Figure 3 summarizes the barriers identified during the study period to using the patients’ reporting of symptoms in each step of the workflow in the clinic. The barriers were identified from the semi-structured interviews with patients and oncologists, the patient evaluation questionnaire and the researcher’s daily registration during the study period.

### System availability

The AmbuFlex software was continuously available, apart from a three-minute offline period during the three months’ study period. This offline period did not influence the project.

## Discussion

This study demonstrates that the collection of self-reported data using PRO-CTCAE, with real-time feedback to treating oncologists employing the AmbuFlex system, is feasible and acceptable in routine clinical practice with male patients undergoing treatment for prostate cancer. Patients stated that the PRO-CTCAE items provided a complete overview of their experienced side effects and that it was a good communication tool. Oncologists found the online platform easy to use and reported that patients were better prepared for the dialogue.

The strength of the study was the sizable percentage of patients who agreed to participate in the study and the substantial number of completions of the PRO-CTCAE questionnaire. The study included male patients only, which could be a limitation. However, we find it plausible that the results, showing good acceptability in the group of elderly men with different social and educational backgrounds, will also be applicable to female adults and other cancer diagnoses, as shown in other studies [9, 11]. Further research is needed to examine the acceptability in other groups.

In our study, the researcher was present in the clinic during the study period, handed over the tablet computer and gave instructions to patients on how to complete the PRO-CTCAE questionnaire. Therefore, the setup did not reflect the routine clinical workflow. If the tested model is to be implemented in routine practice, it will be necessary to provide additional training to patients, oncologists, nurses and secretaries. Our study results indicate that incorporation into local guidelines, including all the above-mentioned matters, and sufficient information to the clinicians are important for facilitating patients’ reporting of symptoms before consultation. The sustainability of the tested model will depend on local resources to cover training, however. Further, the electronic symptom surveillance using PRO-CTCAE has the potential to have alerts be developed, such as for severe symptoms, missed surveys, missed review by clinicians. Such functionalities will be included in future studies.

We also found a high acceptance of the technical facilities, in accordance with other feasibility studies of electronic self-reporting systems [9–12], and patient satisfaction with using the tool when communicating with oncologists about symptoms [23, 24]. In addition, oncologists found the AmbuFlex system useful as a tool to facilitate the clinical dialogue with patients. The AmbuFlex system has been implemented in clinics treating patients diagnosed with afflictions such as prostate cancer, epilepsy, renal failure, sleep disorders and neuromuscular diseases and including more than 13,000 patients [21], and the system allows for a graphic presentation of symptoms, which may facilitate the dialogue during the clinical encounter.

About half of the patients in our study reported that their oncologist had reviewed the PRO data in AmbuFlex before the consultation. The log from AmbuFlex showed that 83% of the patients had their answers reviewed at least once during the study period. Even though reviewing the PRO-CTCAE questionnaires was not compulsory, 50% of the total number of completions were reviewed during the study period. Obtaining reviews of all PRO-CTCAE reports might be facilitated by implementing this procedure in local guidelines.

Some oncologists reported that their own assessment differed from patients’ self-reported symptoms. This conforms to previous research showing discrepancies between the clinician’s report and the patient’s self-reporting of the same symptom [25]. Research indicates that part of the discrepancy between clinician and patient report of symptoms occurs because patients report about any symptom whereas clinicians’ reports are directed toward toxicities attributable to treatment [26]. Patient reporting of symptoms is shown to add information to the clinicians reporting [4]. PRO-CTCAE was developed by the US National Cancer Institute to directly incorporate the patients’ experience into the capture and reporting of symptomatic adverse events in cancer clinical trials [27]. Evaluation shows that patient reporting using PRO-CTCAE may also improve the identification of symptoms at baseline prior to trial initiation, thus allowing for a more precise understanding of the symptomatic toxicities attributable to treatment [28]. Future studies may reveal the impact of PRO-CTCAE on side-effect management, for example.

The US Food and Drug Administration (FDA) states that international collaboration on PRO measures is necessary to secure a common framework for generating and reporting patient-centered data, including symptomatic toxicities, from cancer clinical trials [29]. The need for a disease-specific instrument to capture patients’ symptomatic toxicities according to the relevant therapies has been emphasized [29]. One of the most frequently-used questionnaires on patient-reported symptoms are the quality of life questionnaires of the European Organisation for Research and Treatment of Cancer (EORTC). While health-related quality of life (HRQOL) measures capture a broad picture of the patients’ condition, including family and social factors, they pay less attention to specific toxicity symptoms relating to the individual cancer therapy. PRO-CTCAE has been suggested as a new standard instrument to measure symptomatic toxicities in clinical cancer trials, which is flexibly adaptable to different diagnoses and therapies [29].

One challenge to introducing patient reporting of side effects is the risk of increasing clinicians’ workloads, which has been suggested as a potential barrier [28]. When using PRO-CTCAE feedback as additional information from the patient, oncologists stated that they did not spend extra time on the consultation.

Traditionally, direct patient self-reporting using patient-reported outcome measures has not been systematically incorporated into toxicity reporting. PRO-CTCAE changes this paradigm. As studies have shown high acceptance among patients in the self-reporting of symptoms and that technical barriers can be overcome, the main remaining barrier is to engage clinicians [30], which might be addressed by means of systematic training [31], decision support, and clear accountability metrics. In future studies, special focus must be on clinicians’ engagement in using PROs.

## Conclusions

Electronic patient reporting of symptomatic toxicities using PRO-CTCAE was deemed feasible in prostate cancer patients receiving chemotherapy. Patients and oncologists stated that the electronic system was easy to use and that the patient’s electronic reporting of PRO-CTCAE symptomatic toxicities served to enhance communication. The fact that electronic symptom surveillance with clinician feedback using the Patient-Reported Outcomes version of Common Terminology Criteria for Adverse Events (PRO-CTCAE) in Danish prostate cancer patients was shown to be feasible in this study makes us hypothesize that the model is applicable to future research. Our future research will examine the impact of providing patients’ PRO-CTCAE results to clinicians prior to the visit on the quality of side effects management and resultant clinical outcomes.
